# MicroRNA 139-5p coordinates APLNR-CXCR4 crosstalk during vascular maturation

**DOI:** 10.1038/ncomms11268

**Published:** 2016-04-12

**Authors:** Irinna Papangeli, Jongmin Kim, Inna Maier, Saejeong Park, Aram Lee, Yujung Kang, Keiichiro Tanaka, Omar F. Khan, Hyekyung Ju, Yoko Kojima, Kristy Red-Horse, Daniel G. Anderson, Arndt F. Siekmann, Hyung J. Chun

**Affiliations:** 1 Department of Internal Medicine, Yale Cardiovascular Research Center, Section of Cardiovascular Medicine, Yale University School of Medicine, 300 George Street, 7th Floor, New Haven, Connecticut 06511, USA; 2 Department of Life Systems, Sookmyung Women's University, Seoul 140-742, Korea; 3 Max Planck Institute for Molecular Biomedicine, Roentgenstr. 20, 48149 Muenster, Germany; 4 David H. Koch Institute for Integrative Cancer Research, Massachusetts Institute of Technology, Cambridge, Massachusetts 02142, USA; 5 Department of Biological Sciences, Stanford University, Stanford, California 94305, USA

## Abstract

G protein-coupled receptor (GPCR) signalling, including that involving apelin (APLN) and its receptor APLNR, is known to be important in vascular development. How this ligand–receptor pair regulates the downstream signalling cascades in this context remains poorly understood. Here, we show that mice with *Apln, Aplnr* or endothelial-specific *Aplnr* deletion develop profound retinal vascular defects, which are at least in part due to dysregulated increase in endothelial CXCR4 expression. Endothelial CXCR4 is negatively regulated by miR-139-5p, whose transcription is in turn induced by laminar flow and APLN/APLNR signalling. Inhibition of miR-139-5p *in vivo* partially phenocopies the retinal vascular defects of APLN/APLNR deficiency. Pharmacological inhibition of CXCR4 signalling or augmentation of the miR-139-5p-CXCR4 axis can ameliorate the vascular phenotype of APLN/APLNR deficient state. Overall, we identify an important microRNA-mediated GPCR crosstalk, which plays a key role in vascular development.

## Abstract (web summary)

G protein-coupled receptors APLNR and CXCR4 are crucial for vascular development. Here, the authors show that these two signaling pathways communicate and that in response to blood flow APLNR signaling induces a decrease in CXCR4 expression via miR-139-5p, thereby restricting CXCR4 expression to the non-flow exposed tip cells in the retinal vasculature.

## Graphical abstract

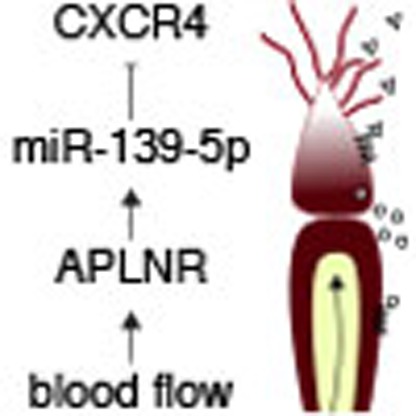

A functional vascular system is fundamental for normal vertebrate development, as it is critical for delivery of oxygen and nutrients, while removing waste to ensure growth and differentiation of all tissues. The concept of tip and stalk cells has emerged over the past decade in the context of sprouting angiogenesis, and guides our understanding of the transition from vascular sprouting to maturation and ultimately quiescence^1^,^2^. In this context, endothelial cell (EC) subsets can respond to molecular and biomechanical cues, and acquire non-proliferative tip cell identities which can extend numerous filopodia and guide vascular outgrowth, or become stalk cells that are considered proliferative and capable of forming lumens^3^. Both the vascular endothelial growth factor (VEGF) and the Notch signalling pathways have been extensively described to regulate the discrimination between these cell populations during mouse and zebrafish development^3^,^4^,^5^,^6^,^7^,^8^. Despite the important role and extensive characterization of the VEGF–Notch signalling paradigm, several additional tip and stalk cell-specific genes have been identified, including APLNR (stalk cells) and CXCR4 (tip cells), whose genetic deletions lead to profound vascular defects^9^,^10^,^11^,^12^,^13^. Moreover, several G protein-coupled receptor (GPCR)-ligand combinations, including stromal cell-derived factor 1 (SDF-1)/CXCR4, S1P/S1PR1 and apelin (APLN)/APLNR, have been shown to play critical roles in vascular development^9^,^10^,^11^,^12^,^14^,^15^,^16^. Nevertheless, unresolved questions remain, and our understanding of the mechanisms by which these signalling processes are integrated remains incomplete.

Here we define a novel mechanism by which signalling pathways involving two of these GPCRs, namely APLNR and CXCR4, are intricately linked in the developing vasculature. We identify robust flow-induced endothelial expression of APLNR in both *in vitro* and *in vivo* models, which in turn leads to marked suppression of endothelial CXCR4 expression. This crosstalk was found to involve a key shear responsive microRNA (miRNA), miR-139-5p, which is positively regulated by APLN/APLNR signalling and directly targets CXCR4 in ECs. Moreover, we demonstrate novel pharmacological strategies to restore this signalling axis that rescue the vascular phenotypes associated with APLN/APLNR deficiency. Overall, these findings provide key mechanistic insights that involve a critical miRNA-based crosstalk between two GPCR signalling cascades, which regulate important early steps in vascular maturation.

## Results

### APLN/APLNR downregulates CXCR4 in developing retinal vessels

**Figure 1:**
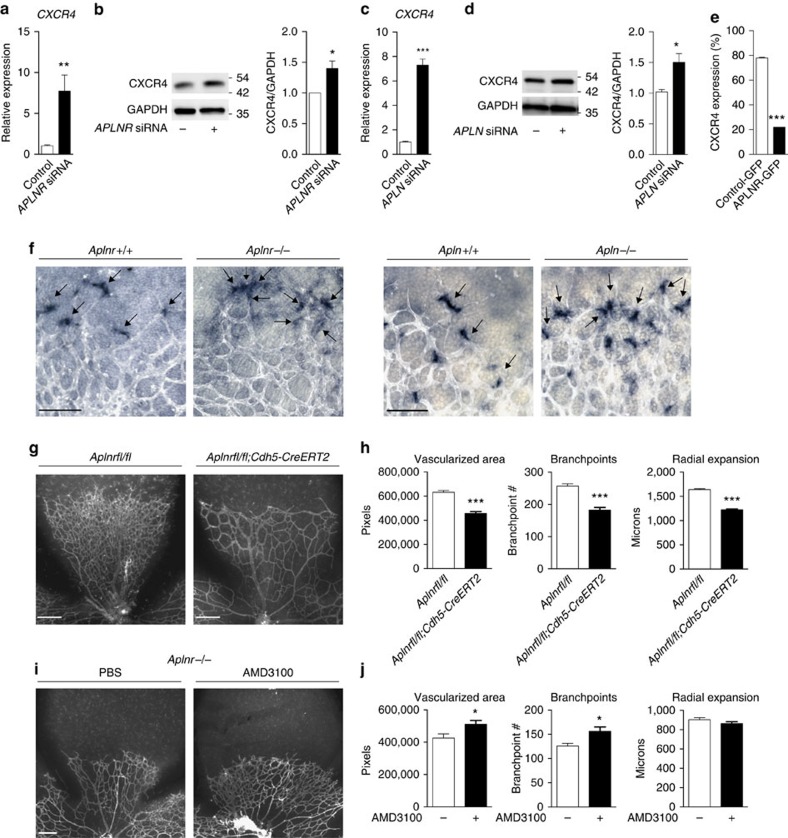
Apelin/APLNR regulates CXCR4 expression *in vitro* and *in vivo*. (**a**–**d**) *CXCR4* transcript and protein levels in response to siRNA-mediated knockdown of *APLNR* (**a**,**b**) or *APLN* (**c**,**d**). *n*=3 experiments per condition. (**e**) Flow cytometry to determine level of CXCR4 expression in HUVECs transfected with either APLNR-GFP or GFP expression constructs. Data shown represents the CXCR4 staining of cells among the GFP-positive cells. *n=*3 experiments. (**f**) *Cxcr4* expression (blue) in P5 retinal vessels as assessed by *in situ* hybridization in *Aplnr*^*−/−*^ and *Apln*^*−/−*^ mice. Arrows indicate *Cxcr4* mRNA localization. Isolectin B4 is shown in white. Scale bar, 100 μm. *n*=8 retinas per genotype. (**g**,**h**) Retinal vasculature of P5 mice with endothelial-specific deletion of *Aplnr* (*Aplnrfl/fl;Cdh5-CreERT2*). Graphs depict vascularized area, branchpoints and radial expansion. Scale bar, 200 μm. *n*≥6 retinas per genotype. (**i**,**j**) Effect of AMD3100 on *Aplnr*^*−/−*^ retinal vasculature. Scale bar, 200 μm. *n*≥5 retinas per genotype. *P<0.05, **P≤0.01, ***P≤0.001, *t*-test. Error bars represent s.e.m.

Our previous investigation of the APLN/APLNR signalling pathway included a gene expression profiling analysis of transcripts that are differentially regulated by *APLN/APLNR* knockdown in ECs (ref. 11). Of the transcripts that were increased, we found that *CXCR4* was one of the most significantly upregulated transcripts in this context (data set GSE46825, Gene Expression Omnibus database (GEO NCBI)). We confirmed that both the messenger RNA (mRNA) and protein levels of CXCR4 are significantly increased in human umbilical vein ECs (HUVECs) subjected to *APLN* or *APLNR* knockdown (Fig. 1a–d). Conversely, flow cytometry analyses demonstrated significant decrease in CXCR4 expression in response to APLNR overexpression in HUVECs (Fig. 1e). To further validate this inverse correlation between APLNR and CXCR4 *in vivo*, we examined mice with genetic deletion of either *Aplnr* or *Apln*. Expression of *Cxcr4* in postnatal day 5 (P5) retinas of both the *Aplnr*^*−/−*^ and *Apln*^*−/−*^ mice, as detected by *in situ* hybridization, was markedly increased compared with respective littermate controls (Fig. 1f). Moreover, to quantitatively assess the changes in endothelial Cxcr4 expression *in vivo*, we carried out flow cytometry analysis using retinas from *Aplnr*^*−/−*^ mice and found significantly higher expression of Cxcr4 in the retinal ECs compared with littermate controls (Supplementary Fig. 1). Both the *Apln*^*−/−*^ and *Aplnr*^*−/−*^ mice also exhibited a significant delay in retinal vascular growth, decreased total vascularized area and vascular branchpoints at P5, as previously demonstrated (Supplementary Fig. 2a,b)^12^,^17^,^18^. To confirm that these phenotypes are specifically due to endothelial APLNR signalling, we investigated the retinal vascular phenotype in mice with conditional, endothelial-specific deletion of *Aplnr* (*Aplnrfl/fl;Cdh5-CreERT2*) using a tamoxifen inducible *Cdh5* Cre driver (*Cdh5-CreERT2*)^19^ (Supplementary Fig. 3a,b). We found that mice lacking *Aplnr* selectively in the endothelium recapitulated the *Aplnr* global knockout phenotype in the retina, albeit less severely (Fig. 1g,h). These mice also exhibited higher levels of Cxcr4 expression in retinal ECs as analysed by flow cytometry (Supplementary Fig. 3c).

Prior studies have shown that loss of CXCR4 signalling results in spindle-like tip cells that fail to migrate properly^10^; however, the effects of excess CXCR4 are not known. To investigate whether the *Apln*^*−/−*^ and *Aplnr*^*−/−*^ vascular phenotypes are in part a consequence of increased CXCR4 signalling, we injected newborn mice with AMD3100, a known selective CXCR4 inhibitor^20^. First, we confirmed that AMD3100 can inhibit effects of SDF-1α in inducing endothelial sprouting in a fibrin gel bead assay (Supplementary Fig. 4). For our *in vivo* studies, as previous studies have demonstrated vascular abnormalities with high doses of AMD3100 (ref. 21), we chose a dose that was subphenotypic in control mice (Supplementary Fig. 5) and examined the retinal vasculature of the *Aplnr*^*−/−*^ mice at P5. We found a significant increase in total vascularized area and vascular branchpoints of the *Aplnr*^*−/−*^ mice treated with AMD3100 compared with the PBS injected controls (Fig. 1i,j); the radial growth of the retinas was not significantly changed, suggesting a partial rescue of the *Aplnr*^*−/−*^ phenotype. Moreover, we also achieved partial rescue of the *Aplnrfl/fl; Cdh5-CreERT2* mice with AMD3100, with increase in total vascularized area and trends towards increase in number of vascular branchpoints and radial growth (Supplementary Fig. 6a,b).

### Endothelial APLNR expression is flow-dependent

**Figure 2:**
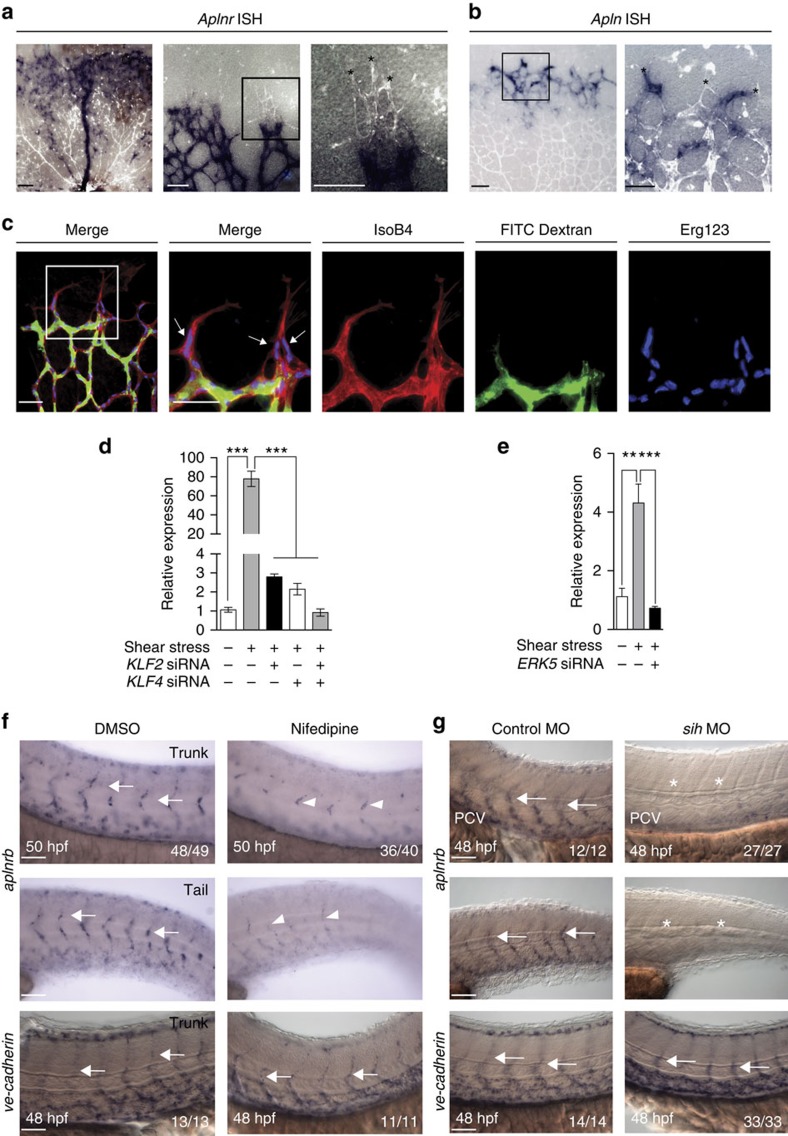
*APLNR* is expressed in newly lumenized vessels and its expression is flow-dependent. (**a**) *Aplnr* expression as determined by *in situ* hybridization (blue) in P5 retinal vasculature with Isolectin B4 staining (white). The boxed area in the middle image is magnified on the right to show higher magnification of the adjacent panel. Scale bar, 50 μm. *n*=3 retinas. (**b**) *Apln* expression as determined by *in situ* hybridization (blue) in P5 retinal vasculature with Isolectin B4 staining (white). The boxed area is magnified on the right to show higher magnification of the adjacent panel. Scale bar, 100 μm (low magnification) or 50 μm (high magnification). *n=*4 retinas. Tip cells are marked by the asterisks. (**c**) Localization of FITC Dextran in the P5 retinal vasculature (green) with Isolectin B4 (red) and Erg123 (endothelial nuclei) staining (blue). The higher magnification images are shown in the right panels. Scale bar, 50 μm. *n=*4 retinas. (**d**) *APLNR* transcript expression in response to shear stress alone or with concurrent *KLF2*, *KLF4* or combined knockdown via siRNA. (**e**) *APLNR* transcript expression in response to shear stress alone or with concurrent *ERK5* knockdown via siRNA. (**f**) *Aplnrb* expression in the trunk and tail of zebrafish embryos at 50 hpf with or without nifedipine (2 h treatment). Arrows (DMSO) and arrowheads (nifedipine) demarcate *aplnrb* expression. (**g**) *Aplnrb* expression in the trunk and tail of zebrafish embryos at 48 hpf with *sih* or control morpholino (MO) injection. Asterisks mark absence of *aplnrb* staining in intersegmental vessels (ISVs). Staining in the posterior cardinal vein (PCV) is also reduced. *In situ* hybridization with the pan-endothelial marker vascular endothelial cadherin (ve-cadherin) shows normal vascular morphology in all conditions. Arrows mark intersegmental blood vessels. Scale bar, 50 μm. **P≤0.01, ***P≤0.001, *t*-test. Error bars represent s.e.m.

We evaluated the retinal vasculature of P5 mice and confirmed that the expression of *Aplnr* was restricted to the stalk cells and maturing veins (Fig. 2a), whereas *Apln* was predominantly expressed by tip cell population (Fig. 2b), as previously reported^12^,^22^. We found that the expression of *Aplnr* was highest in the subset of ECs that surround the newly formed lumen, based on the perfusion pattern observed in fluorescein isothiocyanate (FITC) dextran injected P5 mice (Fig. 2c)^3^. Given the restricted *Aplnr* expression in the retinal vasculature to the leading edge of the lumenized network, which would be the endothelial subset exposed to early onset of blood flow, we evaluated whether shear stress may be a positive regulator of *APLNR* expression. The type of flow in this context has been modelled *in silico*^23^, but remains poorly characterized *in vivo*. We tested the effects of a number of different flow conditions on *APLNR* expression in HUVECs (Supplementary Fig. 7a). Of the different conditions of flow tested, we found the highest upregulation of *APLNR* with orbital shear stress (estimated to induce 10.7 dynes cm^−2^)^24^. We also conducted a serial time course to determine the effect of duration of flow on *APLNR* expression levels, and found that the highest induction was observed at 48 h, while the expression levels began to decrease beyond that time (Supplementary Fig. 7b). Moreover, we found that upregulation of *APLNR* was at least in part dependent on the shear responsive transcription factors KLF2 and KLF4, as knockdown of *KLF2*, *KLF4* or both, markedly inhibited the shear-mediated *APLNR* upregulation (Fig. 2d). In addition, *ERK5* knockdown, a known shear responsive MAP kinase upstream of *KLF2* and *KLF4* (ref. 25), also abrogated induction of *APLNR* expression under shear conditions (Fig. 2e). In contrast to APLNR, APLN expression was significantly downreguated after exposure of HUVECs to shear stress (Supplementary Fig. 8). Lastly, we investigated the role of the second APLNR ligand, namely Toddler (ELABELA)^26^,^27^ and found no effect on CXCR4 expression levels by either its endogenous knockdown or stimulation with exogenous peptide in HUVECs (Supplementary Fig. 9a,b).

To further validate the shear-mediated APLNR regulation *in vivo*, we utilized the zebrafish embryonic system to evaluate the expression of the two paralogous apln receptors, namely *aplnra* and *aplnrb*, under disrupted flow conditions. At baseline, *aplnrb* expression was localized to the intersegmental vessels (ISVs) of 48–50 h post fertilization (hpf) embryos, as determined by *in situ* hybridization (Fig. 2f,g). Following treatment of the embryos with nifedipine, which is known to impede blood flow^28^, or by injection of the *silent heart* (*sih*) morpholino^29^, there was a significant decrease in *aplnrb* expression in the ISVs; demonstrating *in vivo* that blood flow is a critical regulator of *aplnrb* expression (Fig. 2f,g). The ISV vascular structure was unaffected, as confirmed by ve-cadherin staining. *Aplnra* expression in the ISVs, which was at baseline markedly lower than *aplnrb*, was either slightly increased or unchanged by nifedipine treatment or *sih* morpholino injection, respectively (Supplementary Fig. 10a,b).

### APLN/APLNR regulation of CXCR4 is miR-139-5p dependent

**Figure 3:**
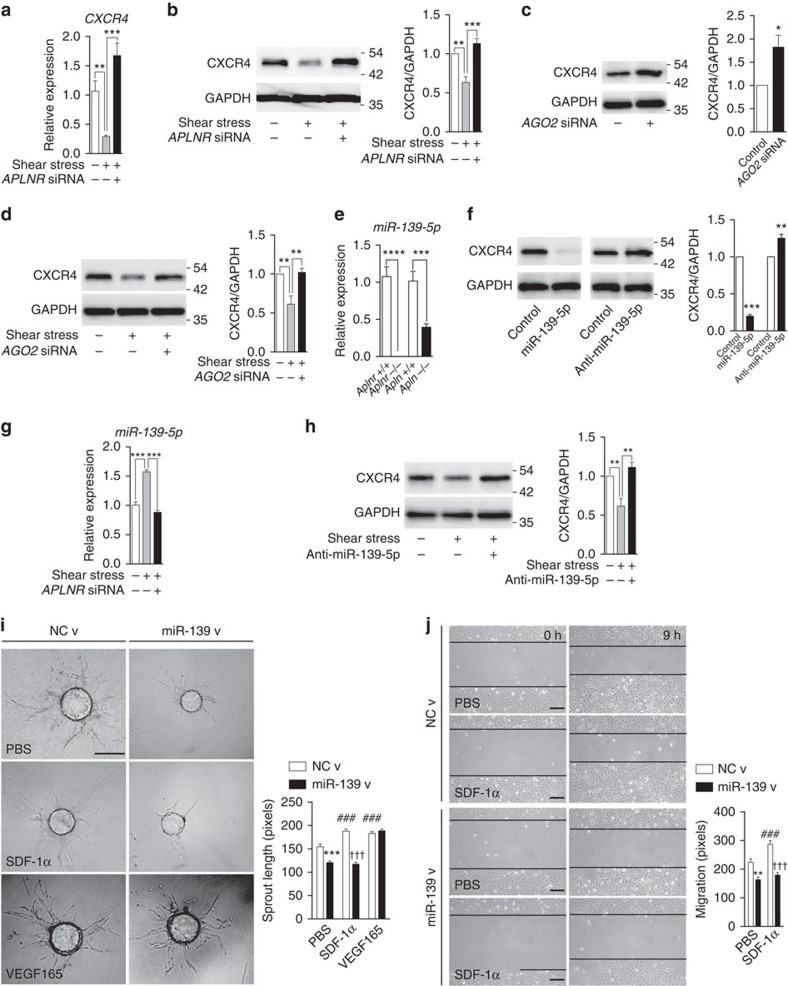
APLNR regulates *CXCR4* expression through miR-139-5p. (**a**,**b**) CXCR4 expression in response to shear stress exposure in HUVECs, with or without concurrent *APLNR* knockdown. mRNA (**a**) and protein levels (**b**) are shown. (**c**) CXCR4 expression in response to *AGO2* knockdown. (**d**) CXCR4 expression in response to shear stress exposure in HUVECs, with or without concurrent *AGO2* knockdown. (**e**) Determination of miR-139-5p levels in FACS sorted ECs from P5 retinas of *Aplnr*^*−/−*^, *Apln*^*−/−*^ mice and respective littermates. *n=*3 retinas per genotype. (**f**) CXCR4 expression in HUVECs overexpressing miR-139-5p mimic or non-targeting control anti-miR. (**g**) Expression levels of miR-139-5p in response to shear stress, with or without concurrent *APLNR* knockdown. (**h**) CXCR4 expression in HUVECs in response to shear stress exposure, with or without concurrent miR-139-5p inhibition via anti-miR. (**i**) Sprouting assay using HUVEC covered beads transduced with miR-139-5p or control lentivirus in response to SDF-1α or VEGF 165. Scale bar, 175 μm. (**j**) Migration assay of HUVECs transduced with miR-139-5p or control lentivirus in response to SDF-1α. Scale bar, 200 μm. *P<0.05, **P≤0.01, ***P≤0.001, ^###^*P*≤0.001 against NC v/PBS, ^†††^*P*≤0.001 against NC v/SDF-1α, *t*-test. Error bars represent s.e.m. *n=*3 experiments per condition.

Previous studies have demonstrated that shear stress can inhibit endothelial CXCR4 expression^30^. We found that the shear-induced downregulation of CXCR4 was abrogated by concurrent knockdown of *APLNR* (Fig. 3a,b), suggesting that APLNR signalling is a critical mediator of flow-induced CXCR4 inhibition. Given the inverse relation between endothelial expression of APLNR and CXCR4, we sought to determine the mechanism by which APLNR regulates CXCR4 expression. Multiple mechanisms are known to affect CXCR4 expression, including emerging data suggesting an important role for miRNA-mediated regulation in different contexts such as breast^31^, prostate^32^, colon^33^ and thyroid^34^ cancer. The role of miRNAs as potential regulators of CXCR4 expression in ECs has not been thus far investigated. We first determined whether miRNAs in general may be playing a role in regulation of CXCR4 in ECs. We subjected HUVECs to *Argonaute 2* (*AGO2*) knockdown, a key protein of the RNA-induced silencing complex, and found a significant increase in CXCR4 expression, suggesting that basal expression of CXCR4 may be miRNA regulated (Fig. 3c). We additionally found that shear-induced downregulation of CXCR4 was also abrogated by concurrent knockdown of *AGO2* (Fig. 3d), demonstrating that a miRNA-based mechanism may be critical in the regulation of CXCR4 expression in the maturing vasculature.

We next set out to identify the miRNA that may be instrumental in APLN/APLNR-mediated CXCR4 regulation. We previously conducted a miRNA expression profiling array using ECs subjected to *APLN* and/or *APLNR* knockdown, with identification of multiple miRNAs that are differentially regulated in this context^35^. Of the miRNAs that were identified in this setting, one in particular, namely miR-139-5p, is predicted to bind the 3′ untranslated region (UTR) of CXCR4 at two independent loci (Supplementary Fig. 11a,b), and was significantly downregulated by disruption of endothelial APLN/APLNR signalling^35^. To further investigate these relationships *in vivo*, we evaluated the expression levels of miR-139-5p in isolated retinal ECs from the eyes of P5 *Apln*^*−/−*^ and *Aplnr*^*−/−*^ mice. We found significantly decreased miR-139-5p levels in fluorescence-activated cell-sorted retinal ECs from *Apln*^*−/−*^ and *Aplnr*^*−/−*^ mice compared with wild-type littermates (Fig. 3e). To further elucidate the association between miR-139-5p and APLN/APLNR signalling, we evaluated the recently described *Aplnr-CreERT; mTmG* mice^36^, which express green fluorescent protein (GFP) driven by the *Aplnr* promoter. Expression of miR-139-5p *in vivo* was significantly enriched in GFP+ retinal cells at P5, further demonstrating the association between APLN/APLNR signalling and miR-139-5p expression (Supplementary Fig. 12). In addition, we found that *APLNR* knockdown decreases the transcripts of both PDE2A, the host gene for miR-139-5p, as well as pri-miR-139, the primary transcript of miR-139-5p (Supplementary Fig. 13), suggesting a direct transcriptional relationship between *APLNR* and miR-139-5p. We next tested the efficacy of miR-139-5p in regulating endothelial CXCR4 expression. Overexpression of miR-139-5p in HUVECs resulted in significant decrease in the protein levels of CXCR4 (Fig. 3f). Conversely, we found that inhibition of miR-139-5p via an anti-miR inhibitor resulted in increased CXCR4 protein levels (Fig. 3f). Moreover, a luciferase reporter activity containing the wild-type CXCR4 3′ UTR was significantly decreased by mir-139-5p overexpression in HUVECs, whereas the same construct with mutagenized miR-139-5p binding sites was unaffected (Supplementary Fig. 14).

### Endothelial CXCR4 is closely regulated by miR-139-5p

To further elucidate the relevance of our findings to the developing vascular context, we examined whether there is a relationship between shear stress and miR-139-5p. We found that miR-139-5p was significantly induced by exposure of ECs to shear stress (Fig. 3g). Moreover, upregulation of miR-139-5p in response to shear exposure was abrogated by concurrent knockdown of *APLNR*, demonstrating the key role of APLNR in mediating shear response in ECs (Fig. 3g). In addition, shear-stress-mediated decrease in CXCR4 expression was abrogated by concurrent inhibition of miR-139-5p (Fig. 3h).

We carried out additional functional studies to investigate the role of miR-139-5p in ECs. Sprouting of ECs in response to SDF-1α was significantly reduced in ECs overexpressing miR-139-5p, whereas sprouting in response to VEGF 165 was unaffected (Fig. 3i). Similarly, HUVECs transfected with an anti-miR against miR-139-5p sprouted more efficiently in response to SDF-1α compared with control transfected cells, while VEGF-induced sprouting was not affected by anti-miR transfection (Supplementary Fig. 15a). Moreover, cell migration in response to SDF-1α was also significantly impeded in HUVECs overexpressing miR-139-5p (Fig. 3j), whereas HUVECs transfected with anti-miR-139-5p had enhanced migration to SDF-1α stimulation (Supplementary Fig. 15b). The migratory response to VEGF 165 was not affected by anti-miR-139-5p (Supplementary Fig. 15b).

**Figure 4:**
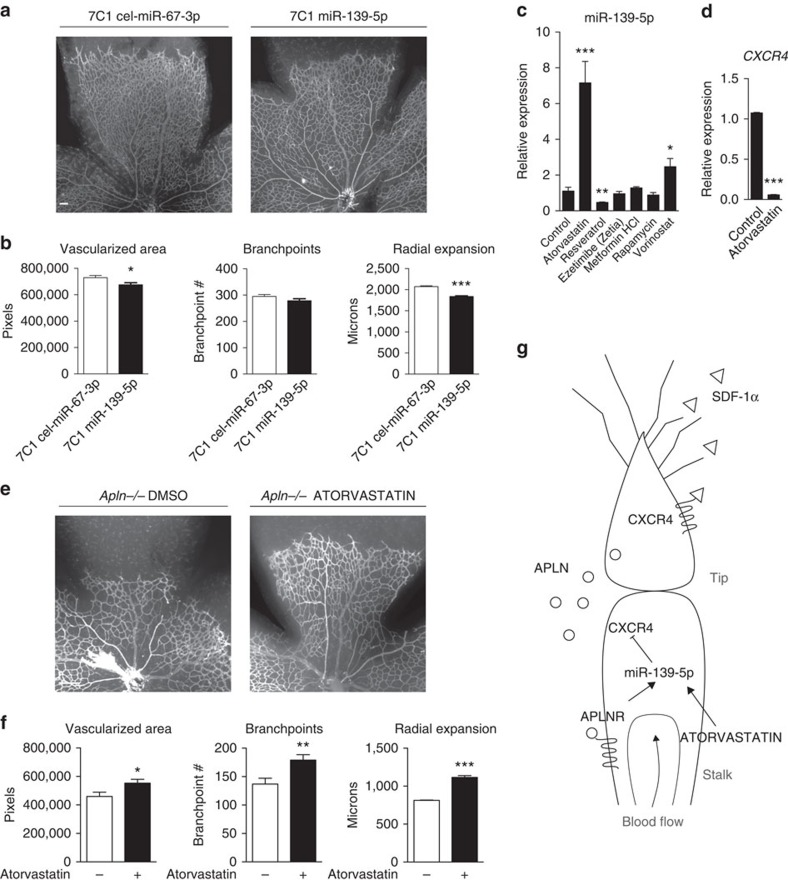
Atorvastatin augments apelin/APLNR signalling and improves the *Apln*^*−/−*^ retinal phenotype. (**a**,**b**) *In vivo* inhibition of miR-139-5p postnatally with 7C1 nanoparticles loaded with anti-miR to miR-139-5p results in reduced vascularized area and radial expansion of retinal vasculature at P5. *n=*6 retinas per genotype. (**c**) Drug screen using clinically approved compounds that affect miR-139-5p expression levels in HUVECs. *n=*3 experiments. (**d**) CXCR4 mRNA expression in HUVECs in response to atorvastatin stimulation. *n=*3 experiments. (**e**,**f**) P5 retinal phenotype of *Apln^−/−^* pups in response to treatment with atorvastatin. Vascularized area, vascular branchpoints and radial expansion are shown. Scale bar, 200 μm. *n*≥4 retinas per genotype. (**g**) Schematic representation of the proposed signalling cascade between APLNR, CXCR4 and the respective ligands in the developing vasculature. *P<0.05, **P≤0.01, ***P≤0.001, *t*-test. Error bars represent s.e.m.

Next, we further validated our findings *in vivo*, using the recently described 7C1 nanoparticles that are capable of selectively targeting oligonucleotide delivery to the endothelium^37^. Intravenous injection of anti-miR-139-5p loaded 7C1 nanoparticles led to partial phenocopying of the *Apln*^*−/−*^ and *Aplnr*^*−/−*^ phenotype, with significantly reduced vascularized area and radial expansion and trend towards reduced number of branchpoints, compared with mice injected with 7C1 loaded with non-targeting control (cel-miR-67-3p; Fig. 4a,b).

### Restoring miR-139-5p-CXCR4 axis rescues *Apln* ^*−/−*^ phenotype

To further validate the role of miR-139-5p as a key downstream element of APLN/APLNR signalling in vascular maturation, we sought to identify a pharmacological strategy to restore its endothelial expression. We screened multiple Food and Drug Administration (FDA) approved compounds to determine whether any of them can induce miR-139-5p expression. Of the six compounds examined, we found that atorvastatin, belonging to the class of 3-hydroxy-3-methyl-glutaryl-CoA reductase inhibitors^38^, induced the highest increase in miR-139-5p expression in HUVECs (Fig. 4c). We further investigated whether atorvastatin can regulate CXCR4 expression; we found that treatment of HUVECs with atorvastatin led to marked inhibition of CXCR4 expression (Fig. 4d).

Our previous studies demonstrated that statins can augment APLN/APLNR signalling, in an APLNR-dependent manner^39^. Given the present data, we sought to investigate whether atorvastatin can potentially rescue the retinal vascular phenotype of mice with *Apln* or *Aplnr* deficiency. We found that atorvastatin treatment of *Apln*^*−/−*^ mice led to robust improvement in retinal vascularized area, radial expansion and increased the number of branchpoints (Fig. 4e,f). The phenotypic rescue of either the *Aplnr*^*−/−*^ or the *Aplnrfl/fl;Cdh5-CreERT2* mice were more modest (Supplementary Fig. 16a,b), likely in part due to the more severe phenotype of these mice, and supporting our previous data that endothelial signalling cascades induced by statin are at least in part APLNR dependent^39^.

## Discussion

Vascular development and maturation is a complex process involving crosstalk among multiple independent, yet closely integrated signalling cascades. VEGF and Notch signalling pathways have been extensively characterized in this context as key factors that provide an intricate orchestration of endothelial identities and promote vascular network formation^2^. A distinct set of signalling pathways whose characterization remains at a relative infancy in this context is that mediated by GPCRs. The importance of GPCR signalling in vascular development is highlighted by the embryonic lethal phenotypes of several GPCRs and their respective ligand knockout mice, described to display a spectrum of cardiovascular defects^9^,^11^,^14^,^40^,^41^,^42^,^43^. However, their downstream targets and interacting partners remain incompletely defined. Adding to the complexity are emerging studies that demonstrate GPCR activation by mechanical forces such as cellular stretch and shear stress in a ligand-independent manner, which may in part explain the phenotypic differences between ligand and receptor knockout mice^44^,^45^,^46^.

CXCR4 is a GPCR with widespread functional roles in a number of contexts, including angiogenesis, hematopoiesis, neurogenesis and immune function^47^. In the context of vascular development, it has been established as a tip cell-specific gene in the developing mouse and zebrafish vasculature^10^,^48^. Interestingly, it is one of a few tip cell enriched endothelial genes that is also known to be markedly downregulated by shear stress *in vitro*^30^, and by blood flow in the zebrafish *in vivo*^48^,^49^. These findings suggest that CXCR4 needs to be strictly regulated in the developing vasculature, to restrict the cellular response to its ligands such as SDF-1α, and avoid the potential detriment of inappropriate EC behaviour resulting from aberrantly expressed CXCR4. Although this possibility has not been specifically examined in the vascular context, whole embryo overexpression of Cxcr4a in zebrafish results in significant craniofacial and cartilage defects, highlighting the requirement for stringent CXCR4 regulation^50^. In addition, although multiple studies have suggested pro-angiogenic effects of augmenting CXCR4 signalling^49^,^51^,^52^, its restricted expression pattern in the developmental context^10^,^48^ suggests the importance of limiting expression to endothelial subsets for normal angiogenesis. Mechanisms that regulate its expression, however, remain inadequately defined. The current body of work provides key insights into a novel mechanism that integrates flow, a secondary GPCR signalling mediated by APLN/APLNR, and miR-139-5p, to restrict endothelial CXCR4 expression in the context of vascular development and maturation (Fig. 4g for schematic).

The abnormal vascular patterning of *Apln* and *Aplnr* null mice likely involves a multitude of downstream signalling targets, some of which have been described previously in other endothelial contexts^11^. Our findings that CXCR4 expression is decreased by APLNR-mediated signalling, and that inhibition of CXCR4 activity with AMD3100 can achieve a partial rescue of the *Aplnr*^*−/−*^ and *Aplnrfl/fl;Cdh5-CreERT2* phenotype highlights the importance of this mechanism, but also implicate other downstream targets that are also likely involved in regulating processes such as cellular proliferation and migration, lumen formation and maintenance, and regulation of vascular permeability^53^. Ongoing studies will further delineate the role of this and other related GPCR signalling cascades in vascular maturation.

Onset of blood flow, and subjecting ECs to shear stress leads to a remarkable alteration in the endothelial behaviour and function. Multiple *in vivo* models have demonstrated the role of flow in vascular development in murine^54^,^55^, avian^56^ and fish models^57^. A number of shear responsive endothelial elements have also been identified, including kinases (ERK5 (ref. 58), ERK1/2 (ref. 59) and AKT (ref. 60)), transcription factors (KLF2 (ref. 61), and KLF4 (ref. 62)) and other epigenetic modifiers (miR-126 (ref. 57)). Our current findings highlight the induction of APLNR expression in ECs subjected to shear stress, an effect that was dependent at least in part on ERK5, KLF2 and KLF4, which are genes with key flow responsive roles in the endothelium.

The specific characterization of the flow pattern in the developing vascular bed remains incompletely defined, although recent studies have used computational analyses to estimate the shear profiles^23^. We found induction of APLNR in ECs in response to multiple types of shear stress, and furthermore showed that *aplnrb*, one of the two paralogous apln receptors in the zebrafish, was markedly decreased with cessation of blood flow.

miRNAs are emerging as vital rheostats in multiple biological processes, and their key roles in the context of vascular development are only beginning to be appreciated. The intricacies of miRNA-mediated signalling integration in vascular development remain at infancy, although recent studies have demonstrated their key roles in angiogenesis^57^. Although miR-139-5p has been described in a limited number of studies as a potential tumour suppressor miRNA^63^,^64^, little is known about the function or regulation of miR-139-5p in the endothelium. Our current findings demonstrate two key aspects of this highly regulated endothelial miRNA that is likely a critical mediator of vascular maturation. First, we identified miR-139-5p as a novel shear responsive endothelial miRNA, that is APLN/APLNR dependent. Second, we found that miR-139-5p is a key intermediary that links APLN/APLNR signalling with repression of endothelial CXCR4 expression, which appears to be essential for proper vascular maturation. Lastly, we show that *in vivo* inhibition of miR-139-5p postnatally results in partial phenocopying of the *Apln*^*−/−*^ and *Aplnr*^*−/−*^ phenotypes. We expect that our ongoing studies into the role of miR-139-5p in the endothelium, both in the developmental and the vascular disease context, will shed greater insights into its functions.

A key aspect of our current findings is the effect of statins in induction of miR-139-5p expression, inhibition of CXCR4 expression and pharmacological rescue of the retinal phenotype of the *Apln*^*−/−*^ mice. These findings provide support for our previous findings describing the augmentation of APLNR-mediated signalling by statins^39^, the mechanism of which will require further investigation. From the perspective of statin pleiotropy, these findings also invoke new potential endothelial targets of statins, which may be playing a key role in mediating the vascular protective effects of these agents in atherosclerosis.

In summary, the crosstalk between APLNR and CXCR4-mediated pathways provide a novel mechanism that may be a crucial determinant of vascular maturation. Further studies to expand on these findings, as well as identification of potential relation between these elements and the role of VEGF–Notch signalling cascade, will advance our understanding of the signalling crosstalk involved in vascular maturation.

## Methods

### Animals

All animal experiments were conducted in compliance with the relevant laws and institutional guidelines and were approved by Yale University Institutional Animal Care and Use Committee and local animal ethics committees of the Landesamt für Natur, Umwelt und Verbraucherschutz Nordrhein-Westfalen. *Aplnr*^*−/−*^, *Aplnrfl/fl*, *Apln*^*−/−*^, *Cdh5-CreERT2* and *Aplnr-CreER; Rosa*^*mTmG*^ mice have been previously described^19^,^36^,^65^. All animals were maintained on a C57Bl/6 background, except for *Aplnr-CreER; Rosa*^*mTmG*^ which were on a mixed background. Given the young age of the mice at the time of our studies, their gender was not determined. To inhibit CXCR4, 5 mg kg^−1^ of AMD3100 (Sigma-Aldrich) dissolved in PBS were injected intraperitoneally in mice at P0 and P1. Injection with PBS was used as negative control. Atorvastatin (Cayman Chemical) was dissolved in dimethyl sulfoxide (DMSO) at 15 mg ml^−1^ and further diluted in PBS. Mice were injected intraperitoneally at P1, P2 and P3 with 40 mg kg^−1^. Injection with PBS/DMSO was used as negative control. To induce recombination of the *Aplnr* allele with the *Cdh5-CreERT2* driver or recombination of *Rosa*^*mTmG*^ with the *Aplnr-CreER* driver, mice received intragastric injection of 100 μg of Tamoxifen (Sigma-Aldrich) dissolved in corn oil (Sigma-Aldrich) at P1 and P2. FITC Dextran (MW=2 × 10^6^ Da; Sigma-Aldrich) was dissolved in water and injected in the left ventricle of anaesthetized P5 pups and allowed to perfuse for 1 min. 7C1 nanoparticles^37^ containing custom made miRIDIAN Hairpin inhibitors for miR-139-5p or cel-miR-67-3p (Dharmacon) were injected daily intravenously at 1 mg kg^−1^ at P0–P2, via the superficial temporal facial vein^66^. All mice for retinal vasculature studies were killed at P5.

For zebrafish blood flow blocking experiments, a stock solution of 10 mM nifedipine (Sigma-Aldrich) in DMSO was diluted 1:4,000 (working concentration 2.5 μM) in E3 medium (diluted from a 60 × stock containing 34.8 g NaCl, 1.6 g KCl, 5.8 g CaCl_2_ × 2H_2_O, 9.78 g MgCl × 6H_2_O dissolved in 2 l H_2_O and pH adjusted to 7.2 with NaOH) containing 2 × tricaine and 0.003% phenylthiourea to prevent pigmentation. Tricaine (2 ×) was prepared by adding 8.4 ml of a stock solution (400 mg tricaine powder, 97.9 ml H_2_O, 2.1 ml Tris (pH9), pH adjusted to 7.0) to 100 ml of E3 medium. Embryos were incubated for 1 h and then fixed in 4% paraformaldehyde (PFA) for *in situ* hybridization experiments. For washout experiments, embryos were washed once with E3 medium, and then transferred to E3 medium without nifedipine.

Morpholinos targeting *cardiac troponin T2* (silent heart (*sih*), 2 ng per embryo) were designed to bind the translation start codon and flanking 5′ sequence (5′-CATGTTTGCTCTGATCTGACACGCA-3′) and injected into embryos at the 1−4 cell stage.

### Immunostaining and *in situ* hybridization

Isolectin B4 staining (Life Technologies, working concentration 20 μg ml^−1^) was performed at room temperature for 1 h or at 4 °C overnight. Erg123 (ab92513, Abcam, 1/100) antibody staining was performed at 4 °C overnight. Alexa Fluor (Life Technologies, working concentration 10 μg ml^−1^) secondary antibody was used. Retinal analysis of the different mice was performed on at least four mice per genotype. Retinas were analysed with a Nikon Eclipse 80i microscope, equipped with a Retiga 2000R Fast1394 camera. NIS Elements software was used for image acquisition and Adobe Photoshop CS5.1 and ImageJ and Matlab were used for image processing. For quantification of vascularized area, vascular branchpoints and radial expansion of the retinas at least five images per retina were acquired at × 20 magnification at the tip-stalk cell border and between arteries and veins.

For *in situ* hybridization eyes from P5 pups were fixed in 4% PFA for 30 min at 4 °C, before the retinas were collected and fixed again in 4% PFA at 4 °C overnight. Retinas were dehydrated in methanol series and stored at −20 °C until required. Before hybridization, retinas were rehydrated to PBS/0.1% Tween and then digested for 11 min in 80 μg ml^−1^ Proteinase K (Sigma-Aldrich) followed by fixation in 4% PFA/0.2% glutaraldehyde in PBS/0.1% Tween. Retinas were washed in PBS/0.1% Tween, pre-incubated in hybridization buffer at 70 °C for 3 h and then incubated with RNA probes in hybridization buffer at 70 °C overnight. Probes were labelled with digoxigenin-uridine triphosphate (Roche) using full-length complementary DNA (cDNA) clones and T7, T3 or Sp6 polymerases. The *Apln* plasmid was a kind gift from A. Eichmann^12^, *Aplnr* and *Cxcr4* plasmids were purchased from Dharmacon (MMM1013-202766118, clone ID: 4457726, MMM1013-202762695, clone ID: 3592479). After extensive washing the hybridized probes were detected with alkaline phosphatase–conjugated anti-digoxigenin-alkaline phosphatase antibody (11093274910, Roche, working concentration 0.375 U ml^−1^) at 4 °C overnight and signal was visualized with BM purple alkaline phosphatase substrate (1144207001, Roche). Retinas were mounted using Vectashield (Vector Labs) and imaged.

For zebrafish *in situ* hybridization, *aplnra* and *aplnrb* probes were amplified from cDNA of 19 somite stage-old embryos using the following primers: *aplnra*-forward 5′-GAAAGGCCCAAGTCACAGAG-3′ and *aplnra*-reverse 5′-GAGTTCACTATCTGATGTCAAACCA-3′, *aplnrb*-forward 5′-GAAAGGCCCAAGTCACAGAG-3′ and *aplnrb*-reverse 5′-GAGTTCACTATCTGATGTCAAACCA-3′. The T7 promotor was added to the 5′ end of the reverse primer in a second round of amplification (T7-*aplnra*-reverse 5′-GTAATACGACTCACTATAGGGAGTTCACTATCTGATGTCAAACCA-3′, T7-*aplnrb*-reverse 5′-GTAATACGACTCACTATAGGGAGTTCACTATCTGATGTCAAACCA-3′)^67^. The *ve-cadherin* probe was synthesized using a plasmid containing a 1,395-bp *cdh5* DNA segment. mRNA was synthesized *in vitro* using digoxigenin-uridine triphosphate (Roche) and T7 or T3 Polymerase (Roche).

### Cells and reagents

HUVECs (Yale VBT Core) were cultured at 37 °C in a 5% CO_2_ incubator in EGM-2 medium (Lonza). For experimental treatments, HUVECs (passages 3–7) were grown to 70-90% confluence. Laminar or pulsatile shear stress was applied to confluent cell cultures at 12 or 12±5 dynes cm^−2^, respectively, using a parallel plate chamber. A surface area of 14 cm^2^ on the HUVEC-seeded slide was exposed to fluid shear stress generated by perfusing culture medium over the cells. The pH of the system was kept constant by gassing with 5% CO_2_/95% air and the temperature was maintained at 37 °C. Orbital shear stress, calculated at 10.7 dynes cm^−2^, was applied to confluent cell cultures seeded in 6-well plates, using an orbital shaker positioned inside an incubator. Orbital shear stress was used for all shear experiments (other than alternate conditions for Supplementary Fig. 7). Transient transfections of plasmids were performed with Fugene HD (Promega) according to the manufacturer's instructions. For gene silencing, short interfering RNAs (siRNAs; Stealth siRNA, Invitrogen) were transfected with RNAiMAX (Invitrogen) according to the manufacturer's instructions. Non-targeting control and anti-miR-139-5p oligonucleotides (Dharmacon) were transfected with RNAiMAX (Invitrogen) according to the manufacturer's instructions. Control and miR-139 lentivirus constructs (System Biosciences) were used to generate lentivirus particles with the Lenti-X HTX Packaging System (Clontech) with Lenti-X Concentrator. For *in vivo* inhibition of miR-139-5p, custom made miRIDIAN Hairpin inhibitors for miR-139-5p or cel-miR-67-3p were used (Dharmacon) for packaging in the 7C1 nanoparticles^37^. To generate 7C1 nanoparticles, polyethyleneimine with a number molecular weight of 600 (PEI600, Sigma-Aldrich) was combined with 200 proof anhydrous ethanol (Koptec) and an epoxide-terminated C15 lipid at a lipid:PEI molar ratio equal to 14:1. The mixture was heated at 90 °C for 48 h before purification was performed with a silica column^37^. To formulate nanoparticles, purified 7C1 was combined with 200 proof ethanol and (1,2-dimyristoyl-sn-glycero-3-phosphoethanolamine-*N*-[methoxy(polyethylene glycol)-2000] (Avanti Polar Lipids) at a 7C1:lipid-polyethylene glycol (PEG) molar ratio equal to 4:1 in a glass syringe. siRNA was dissolved in pH 3 10 mM citrate solution (Teknova) in a separate syringe. The two syringes were connected to a syringe pump and the fluid was pushed through a microfluidic device. The resulting nanoparticles were dialyzed in 1 × PBS and filtered through a 0.22-μm sieve before their size was characterized.

HUVECs were treated for 24 h with atorvastatin (working concentration 10 μM in DMSO), vorinostat (working concentration 10 μM in PBS), resveratrol (working concentration 10 μM in PBS), ezetimibe (working concentration 10 μM in PBS), metformin (working concentration 100 μM in H_2_O) or rapamycin (working concentration 20nM in EtOH; Cayman Chemical) with the corresponding controls.

### Cell migration assay

HUVECs were incubated in 6-well plates in EGM-2 medium overnight. After serum starvation for 6 h with EBM-2 medium, the monolayer of HUVECs was scratched with a universal blue pipette tip and the widths of the scratches in four fields per well were captured using a Zeiss microscope with a × 10 objective. Cells were incubated for 9 h in starvation medium containing PBS and 50 ng ml^−1^ SDF-1α (SRP3252, Sigma-Aldrich). The same fields were captured again after migration. Differences in the widths of scratches before and after migration were calculated. The calculated width is an average of the widths of three measurements taken from the same field. The means and s.e.m. of triplicate wells were calculated. All experiments were conducted at least three times.

### Fibrin gel bead assay

HUVECs were mixed with Cytodex 3 microcarriers (Amersham Pharmacia Biotech) at a concentration of 400 HUVECs per bead in 1 ml EGM-2 medium. Beads with cells were shaken gently every 20 min for 4 h at 37 °C in 5% CO_2_. After incubation, the beads with cells were transferred to a 25 cm^2^ tissue culture flask and left for 12–16 h in 5 ml EGM-2. The following day, these were washed three times with 1 ml EGM-2 and resuspended at a concentration of 200 cell-coated beads per ml in 2.5 mg ml^−1^ fibrinogen (Sigma-Aldrich) containing 0.15 U ml^−1^ aprotinin (Sigma-Aldrich). A total of 500 μl fibrinogen/bead solution was added to 0.625 U of thrombin (Sigma-Aldrich) in one well of a 24-well tissue culture plate. The fibrinogen/bead solution was allowed to clot for 5 min at room temperature and then at 37 °C in 5% CO_2_ for 15 min. EGM-2 containing 50 ng ml^−1^ SDF-1α (SRP3252, Sigma-Aldrich), or 20 ng ml^−1^ VEGF 165 (293-VE, R&D Systems) was used. Fibroblasts were layered on top of the clot at a concentration of 20,000 cells per well. The medium was changed every other day, and SDF-1α and VEGF 165 were added daily.

### 3′ UTR luciferase reporter assay

Human *CXCR4* 3′ UTR reporter construct was purchased from SwitchGear Genomics. For mutant analysis, the sequence 5′-CTGTAG-3′ in the two predicted miR-139-5p binding sites was mutated to 5′-GGATCC-3′. HUVECs were transfected with the luciferase reporter constructs containing the CXCR4 3′ UTR variants, Renilla luciferase and 30 nM of either miR-139-5p mimic or negative control miRNA. At 24 h after transfection, the cells were lysed, and luciferase activity was measured using the Dual-Luciferase Reporter Assay kit (Promega) according to the manufacturer's instructions.

### Flow cytometry

Up to four retinas of wild type or *Aplnr*^*−/−*^ P5 mouse neonates were pooled and incubated for 15 min at 37 °C in EBM-2 containing Collagenase A (1.5 mg ml^−1^, 103578, Roche) and DNAase I (25 μg ml^−1^, 104159, Roche) before cells were dissociated by gentle trituration. Cells were stained with FITC-conjugated anti-CD31 antibody (558738, BD Biosciences, working concentration 5 μg ml^−1^) or APC-conjugated anti-CXCR4 antibody (558644, BD Biosciences, working concentration 2 μg ml^−1^). Stained cells were sorted using a FACSAria flow cytometer (BD Biosciences) or an LSRII flow cytometer (BD Biosciences). When cells were collected, both the negative and positive selections were collected in ice cold PBS and processed for RNA extraction and real-time (RT) PCR as described below. Data quantification was performed with FlowJo 7.6 software.

HUVECs transfected with GFP or APLNR-GFP (Origene) plasmids for 48 h were detached from the culture dish, washed and subsequently resuspended with PBS containing 5% FBS. The cells were incubated at 4 °C for 30 min with phycoerythrin-conjugated CXCR4 antibody (551510, BD Biosciences, working concentration 2 μg ml^−1^). Cells were washed with PBS and CXCR4 expression was analysed by flow cytometry (BD FACScan). Data quantification was performed with FlowJo 7.6 software.

### RNA analysis

Total RNA was extracted using the miRNeasy RNA isolation kit (Qiagen). Purified RNA was reverse transcribed with the iScript cDNA Synthesis Kit (Bio-Rad) or the TaqMan MicroRNA Reverse Transcription Kit (Life Technologies). RT-PCR was performed with TaqMan probes for both genes and miRNAs (Life Technologies). RT-PCR for miR-139-5p detected both the human (hsa-miR-139-5p) and murine (mmu-miR-139-5p) variants. All miRNA data were normalized to the internal control small RNAs *RNU19* or *U6*. For the mRNA samples, ribosomal 18S was used as internal control. Individual RT-PCRs were performed on a CFX96 (Bio-Rad) according to the manufacturer's instructions. TaqMan probes were used for all genes.

### Protein extraction and western blotting

HUVECs were lysed with RIPA lysis buffer (Thermo Scientific) containing Halt Protease and Phosphatase Inhibitor cocktail (Thermo Scientific). Total protein contents of soluble cell lysate were measured using a Micro BCA Protein assay kit (Thermo Scientific). Western blotting was performed using standard methods. Briefly, total protein from each sample was resolved by sodium dodecyl sulfate–polyacrylamide gel electrophoresis, transferred to a polyvinylidene difluoride membrane (Bio-Rad) and probed with antibodies specific to CXCR4 (ab2074, Abcam, 1:2,000) and GAPDH (2118, Cell Signaling, 1:3,000). Subsequently, the membranes were incubated with HRP-conjugated secondary antibodies (7074, Cell Signaling, 1:3,000) and developed using an enhanced chemiluminescence detection method (Thermo Scientific). Uncropped scans of key western blots are provided as a Supplementary Fig. 17.

### Statistical analyses

Unless otherwise noted, all cell treatments, cell migration and sprouting assays, western blots, quantitative PCR assays and *in situ* hybridizations are representative of at least three independent experiments. Results are reported as mean±s.e.m. Unpaired Student's *t*-test was used to determine statistical significance. *Post hoc* analysis was performed using the Holm–Sidak method. *P*<0.05 was considered significant.

## Additional information

**How to cite this article:** Papangeli, I. *et al*. MicroRNA 139-5p coordinates APLNR-CXCR4 crosstalk during vascular maturation. *Nat. Commun.* 7:11268 doi: 10.1038/ncomms11268 (2016).

## Supplementary Material

Supplementary Information Supplementary Figures 1-17
