# Supplementary material for: MicroRNA 139-5p coordinates APLNR-CXCR4 crosstalk during vascular maturation
Source: Nat Commun. 2016 Apr 12;7:11268. doi: 10.1038/ncomms11268 (PMC4832062; doi:10.1038/ncomms11268)
Supplement: Supplementary Information — Supplementary Figures 1-17 [file ncomms11268-s1.pdf]

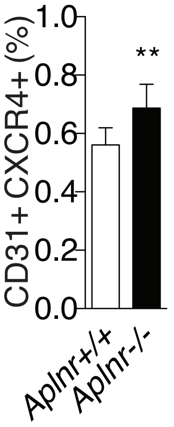

Supplementary Fig. 1. *APLNR* regulates *CXCR4* expression levels in vivo. Flow cytometry sorted endothelial cells from *Aplnr-/-* retinas at P5 have increased levels of *CXCR4* compared to littermate controls. ** $P \leq 0.01$ *t*-test. Error bars represent SEM. $n \geq 4$ retinas per genotype.

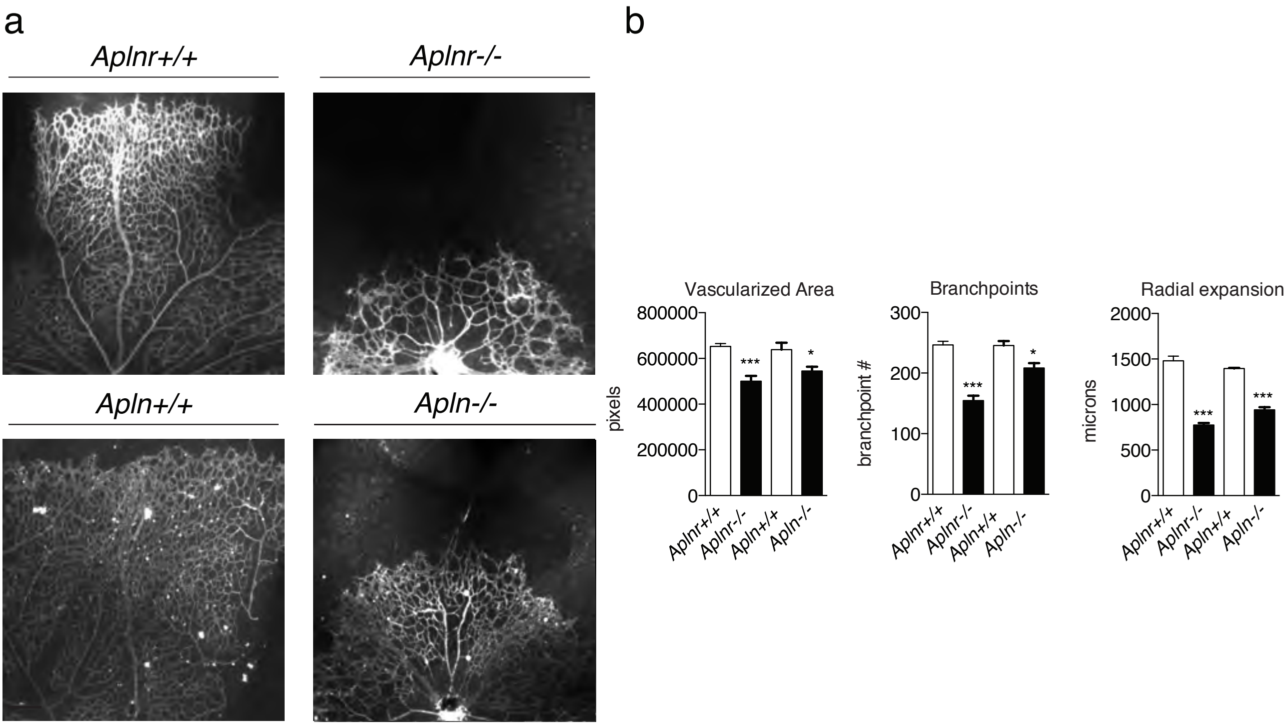

Supplementary Fig. 2. Loss of *Aplnr* or *Apln* leads to retinal angiogenic defects. (a) Retinal vasculature of P5 *Aplnr-/-* or *Apln-/-* mice at P5 which display (b) reduced vascularized area, fewer vascular branchpoints and impaired vascular growth compared to littermate wild type controls. Scale bar = 200 μm. * $P < 0.05$, *** $P \leq 0.001$, *t*-test. Error bars represent SEM. $n \geq 5$ retinas per genotype.

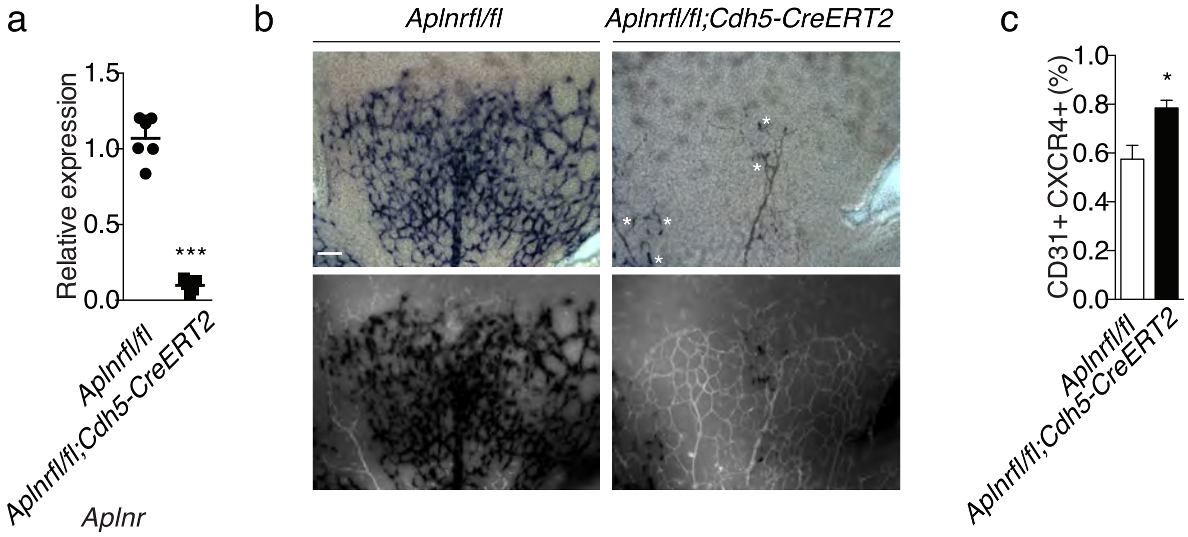

Supplementary Fig. 3. Postnatal deletion of *Aplnr* in the endothelium using a tamoxifen inducible *Cdh5-CreERT2* driver. (a) Whole lung lysates from P5 *Aplnrfl/fl;Cdh5-CreERT2* pups induced with tamoxifen at P1 and P2 have significantly reduced levels of *Aplnr* compared to whole lung lysates from littermate control *Aplnrfl/fl* pups that also received tamoxifen. $n \geq 5$ retinas per genotype. (b) *In situ* hybridization for *Aplnr* shows robust stalk cell and vein specific expression in the P5 mouse retina of tamoxifen injected *Aplnrfl/fl* mice, whereas almost no cells express *Aplnr* in tamoxifen injected *Aplnrfl/fl;Cdh5-CreERT2* mice. Asterisks indicate incomplete recombination in isolated endothelial cells in *Aplnrfl/fl;Cdh5-CreERT2* retinas. n=2 retinas per genotype. (c) Flow cytometry sorted endothelial cells from *Aplnrfl/fl;Cdh5-CreERT2* retinas at P5 have increased levels of *CXCR4* compared to littermate controls. $n \geq 5$ retinas per genotype. Scale bar = 100 μm. * $P < 0.05$, *** $P \leq 0.001$, *t*-test. Error bars represent SEM.

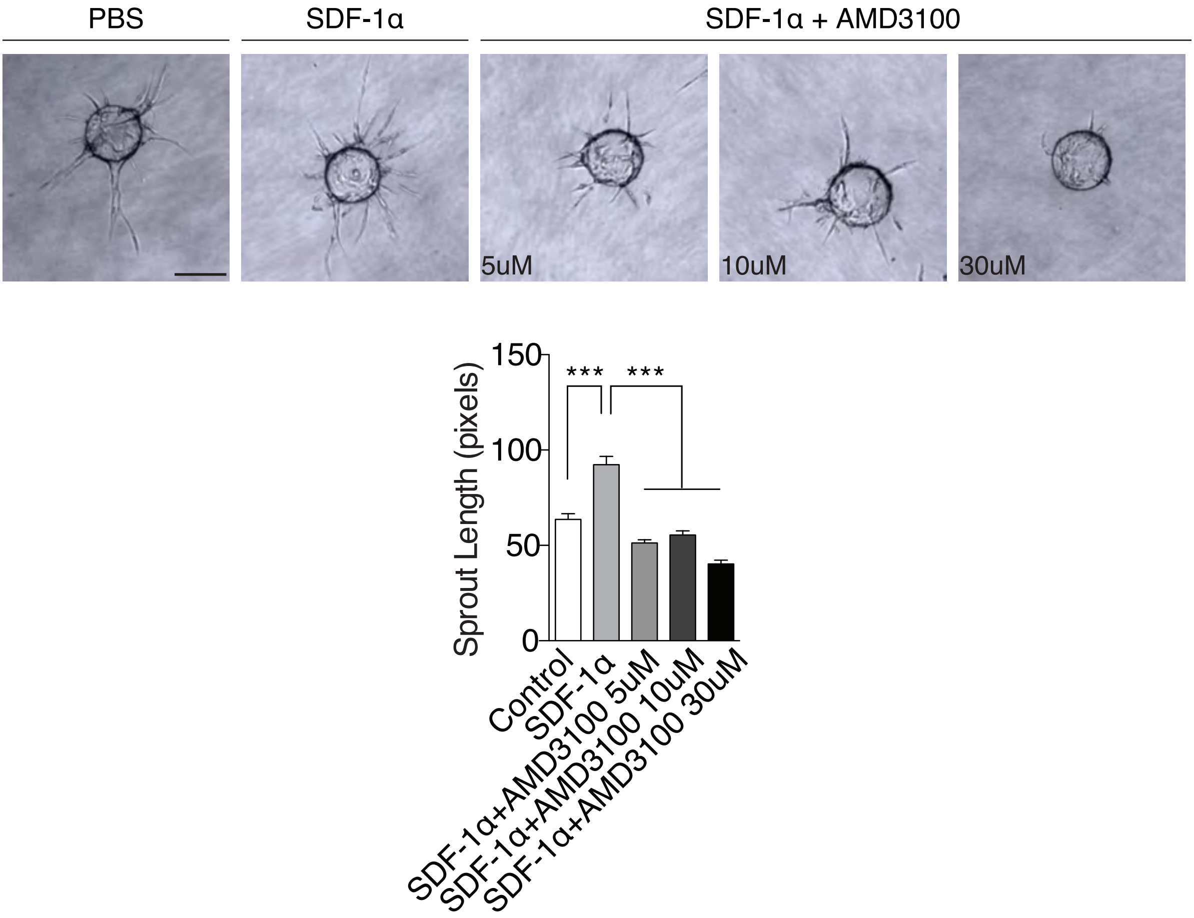

Supplementary Fig. 4. AMD3100 inhibits SDF-1α induced endothelial cell sprouting in a dose dependent manner. Beads covered with HUVECs show reduced sprouting in response to SDF-1α when co-treated with AMD3100. Scale bar = 175 μm. ***$P \leq 0.001$, *t*-test. Error bars represent SEM. *n*=3 experiments per condition.

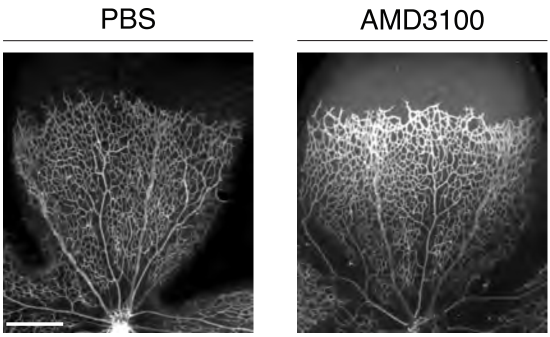

Supplementary Fig. 5. AMD3100 did not have an effect on wildtype retina development at 5mg kg^{-1}. At P5 wildtype mice treated with AMD3100 displayed comparable retinal vascular patterning shown by isolectin B4 staining. Scale bar = 50 μm. *n*=5 retinas per condition.

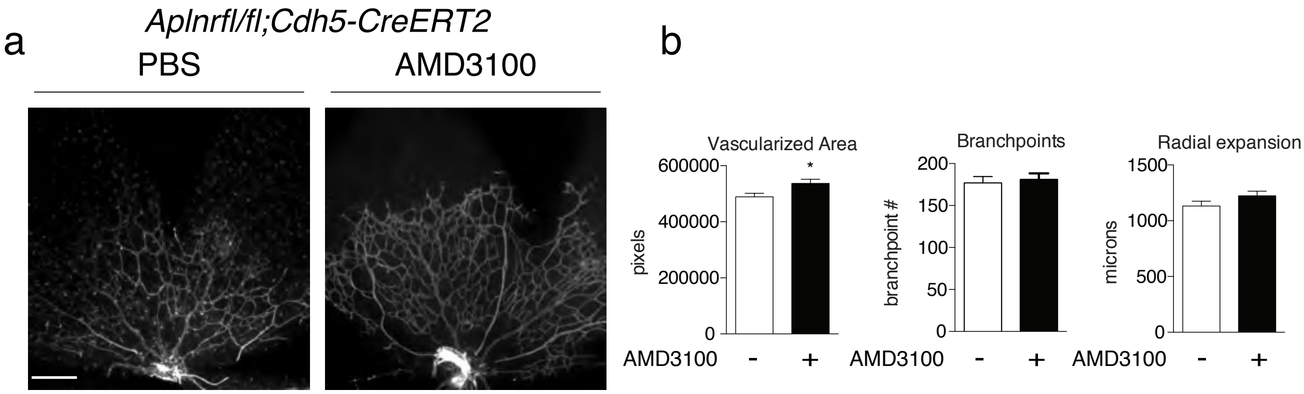

Supplementary Fig. 6. Effect of AMD3100 on *Aplnrfl/fl;Cdh5-CreERT2* retinal vasculature. (a) Retinal vasculature of P5 mice with endothelial specific deletion of *Aplnr* (*Aplnrfl/fl;Cdh5-CreERT2*) treated with AMD3100. (b) Graphs depict vascularized area, number of branchpoints, and radial expansion. Scale bar = 200 µm. * $P < 0.05$, *t*-test. Error bars represent SEM. $n \geq 5$ retinas per genotype.

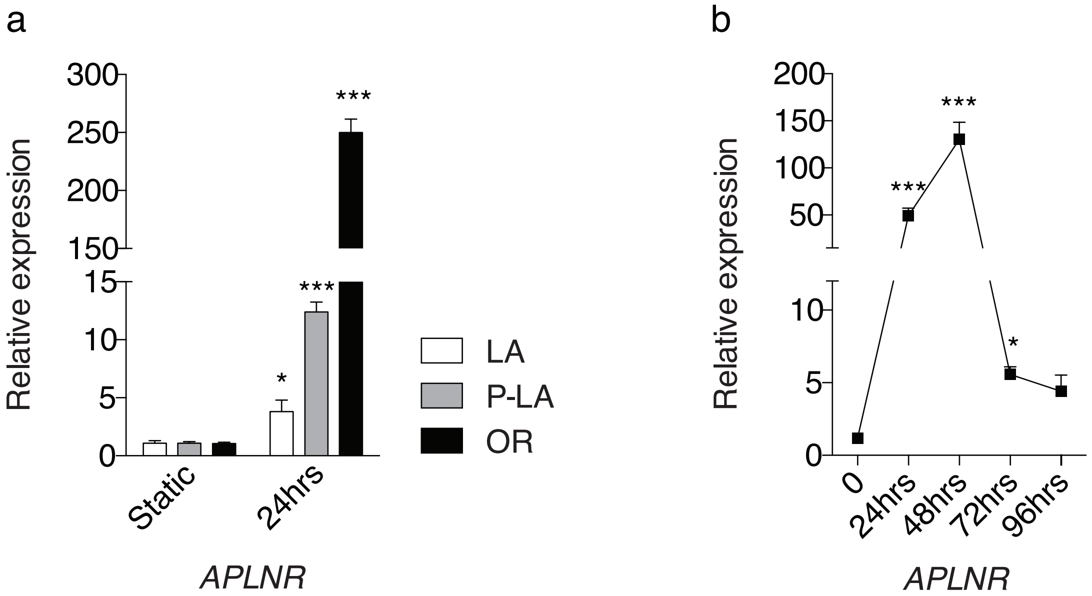

Supplementary Fig. 7. *APLNR* expression is shear stress dependent. Real time PCR showing *APLNR* mRNA expression in cells exposed to (a) 24 hours of laminar (LA, 12 dynes cm^{-2}), pulsatile laminar (P-LA, 12±5 dynes cm^{-2}) or orbital (OR, 10.7 dynes cm^{-2}) shear stress, or (b) 24-96 hours of OR shear stress, compared to static controls. *$P < 0.05$, ***$P \leq 0.001$, *t*-test. Error bars represent SEM. *n*=3 experiments per condition.

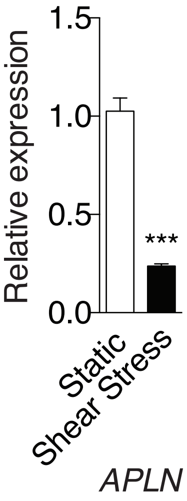

Supplementary Fig. 8. Endothelial *APLN* expression is downregulated by shear stress. Real time PCR showing *APLN* expression levels in cells exposed to 24 hours of orbital (10.7 dynes/cm^2) shear stress compared to static controls. *** $P \leq 0.001$, *t*-test. Error bars represent SEM. *n*=3 experiments per condition.

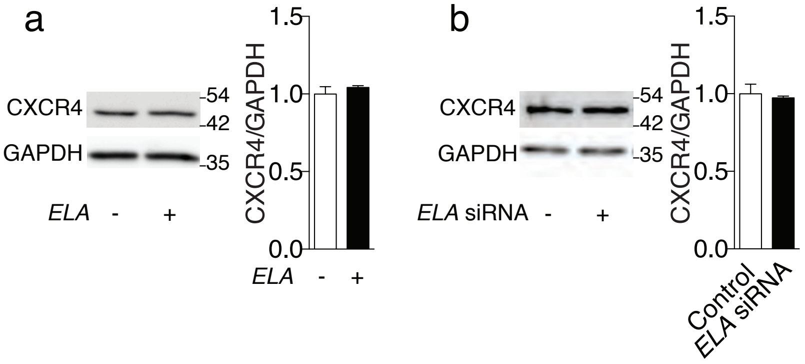

Supplementary Fig. 9. ELABELA does not regulate CXCR4 expression. (a) Stimulation of HUVECs with ELABELA (ELA) or (b) knockdown of *ELA* has no effect on CXCR4 expression. *P*=n.s., *t*-test. Error bars represent SEM. *n*=3 experiments per condition.

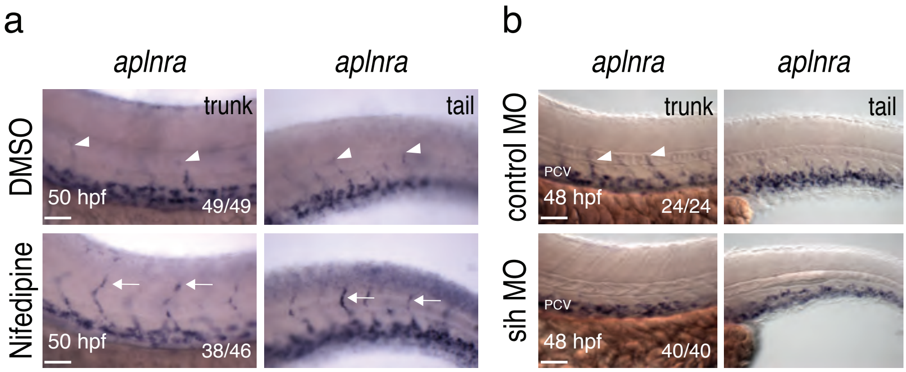

Supplementary Fig. 10. Expression of *aplnra* in zebrafish embryos under distrubed flow conditions. (a) *Aplnra* expression in the trunk and tail of zebrafish embryos at 50 hpf with or without nifedipine (2 hour treatment). Arrowheads (DMSO) and arrows (nifedipine) demarcate *aplnra* expression. (b) *Aplnra* expression in the trunk and tail of zebrafish embryos at 48 hpf with *sih* or control MO injection appears unchanged in the posterior cardinal vein (PCV). Absence of intersegmental vessel expression of *aplnra* (arrowheads) in *sih* MO embryos is likely due to a known defect in vein sprouting in *sih* MO injected embryos. Scale bar = 50 μm.

a

```
5'  UCUACAGUGCACGUGUCUCCAGU  3' hsa-miR-139-5p
    ||||||||||||||||||||||
5'  UCUACAGUGCACGUGUCUCCAG   3' mmu-miR-139-5p
    |||||||||||:||||||
5'  UCUACAGUGCAUGUGUCU       3' dre-miR-139-5p
```

b

```
    3'  gaccucugugCA-CGUGACAUCu  5' hsa-miR-139-5p
                  || | |||||||
230:5'  augucucgugGUAGGACUGUAGa  3' CXCR4

    3'  gaccUCUGUGC--ACGUGACAUCu  5' hsa-miR-139-5p
            ||||::|  | ::||||||
364:5'  cuuaAGACGUGAUUUUGCUGUAGa  3' CXCR4
```

Supplementary Fig. 11. MiR-139-5p is highly conserved among species and is predicted to bind CXCR4 mRNA. (a) Alignment of the mature sequences of miR-139-5p in human, mouse and zebrafish. (b)Two predicted binding sites for miR-139-5p in the human CXCR4 3' untranslated region (UTR).

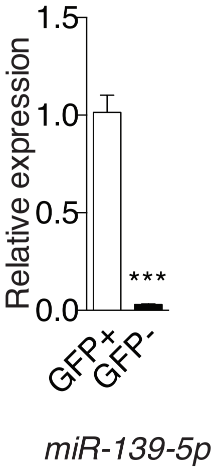

Supplementary Fig. 12. MiR-139-5p is enriched in the *Aplnr* expressing cell population of the developing mouse retina. Real time PCR showing relative transcript levels of miR-139-5p in flow sorted *AplnrCreERT;mTmG* GFP positive and GFP negative retinal cells at P5. ***$P \leq 0.001$, *t*-test. Error bars represent SEM. *n*=2 GFP+ and *n*=3 GFP- retinas.

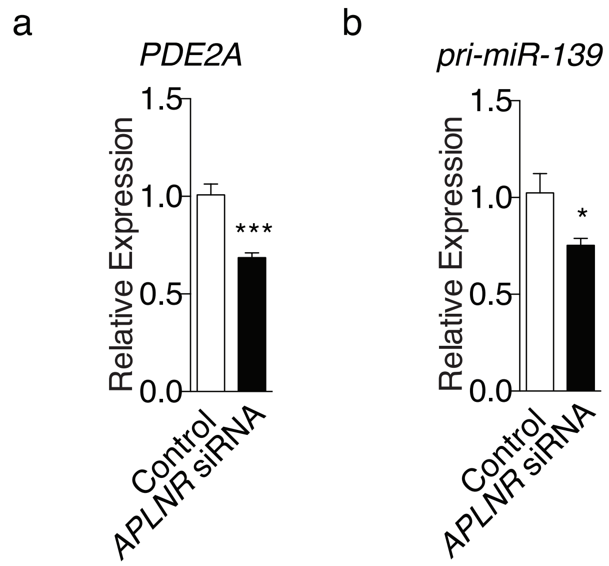

Supplementary Fig. 13. Apelin/APLNR regulates transcription of *miR-139-5p*. Knockdown of *APLNR* induces downregulation of (a) *PDE2A*, which hosts *miR-139-5p* and (b) the primary trasncript of *miR-139-5p*, *pri-miR-139*. * *P* value ≤ 0.05, *** P value ≤ 0.001, *t*-test. Error bars represent SEM. *n*=3 experiments per condition.

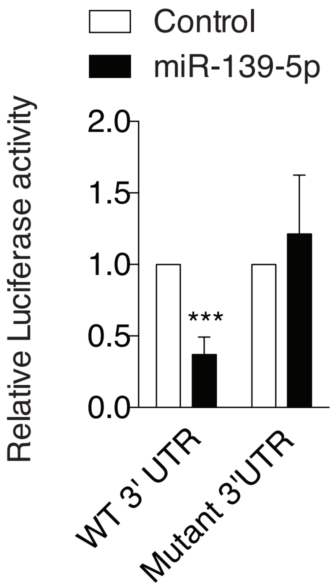

Supplementary Fig. 14. MiR-139-5p targets the two predicted binding sites in the *CXCR4* 3' UTR. A luciferase reporter construct containing the wildtype *CXCR4* 3' untranslated region (UTR) is targeted by miR-139-5p, while a luciferase reporter construct containing the mutant *CXCR4* 3' UTR is unaffected by miR-139-5p overexpression, in HUVECs. $^{***}P \leq 0.001$, *t*-test. Error bars represent SEM. n=3 experiments per condition.

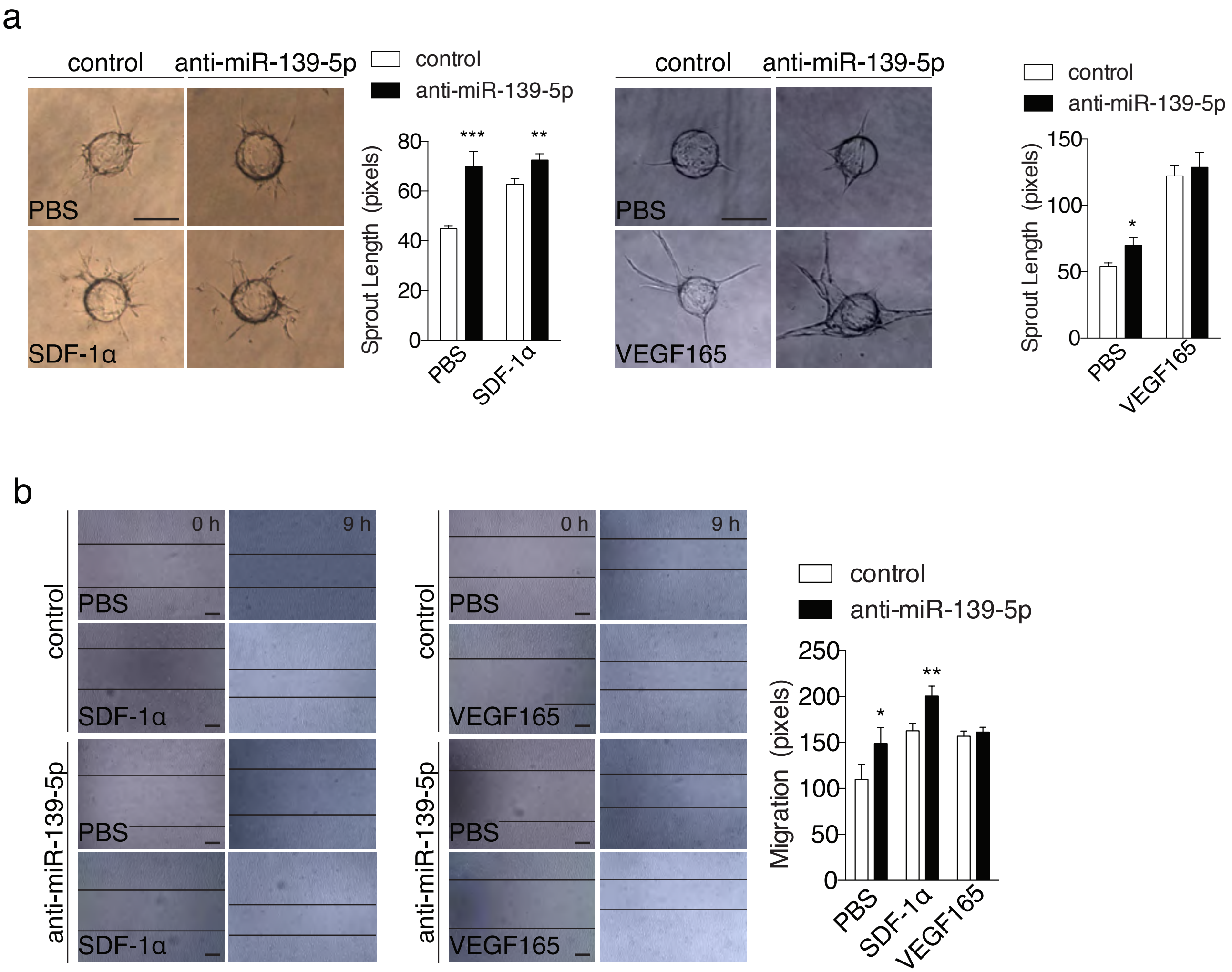

Supplementary Fig. 15. MiR-139-5p targets *CXCR4* and controls angiogenic processes*.* (a) Sprouting assay using HUVEC covered beads transfected with anti-miR-139-5p or non-targeting control anti-miR in response to SDF-1α or VEGF165. Scale bar = 175 μm. (b) Migration assay of HUVECs trasnfected with anti-miR-139-5p or non-targeting control anti-miR in response to SDF-1α or VEGF165. Scale bar = 200 μm. * $P < 0.05$, ** $P \leq 0.01$, *** $P \leq 0.001$, *t*-test. Error bars represent SEM. *n*=3 experiments per condition.

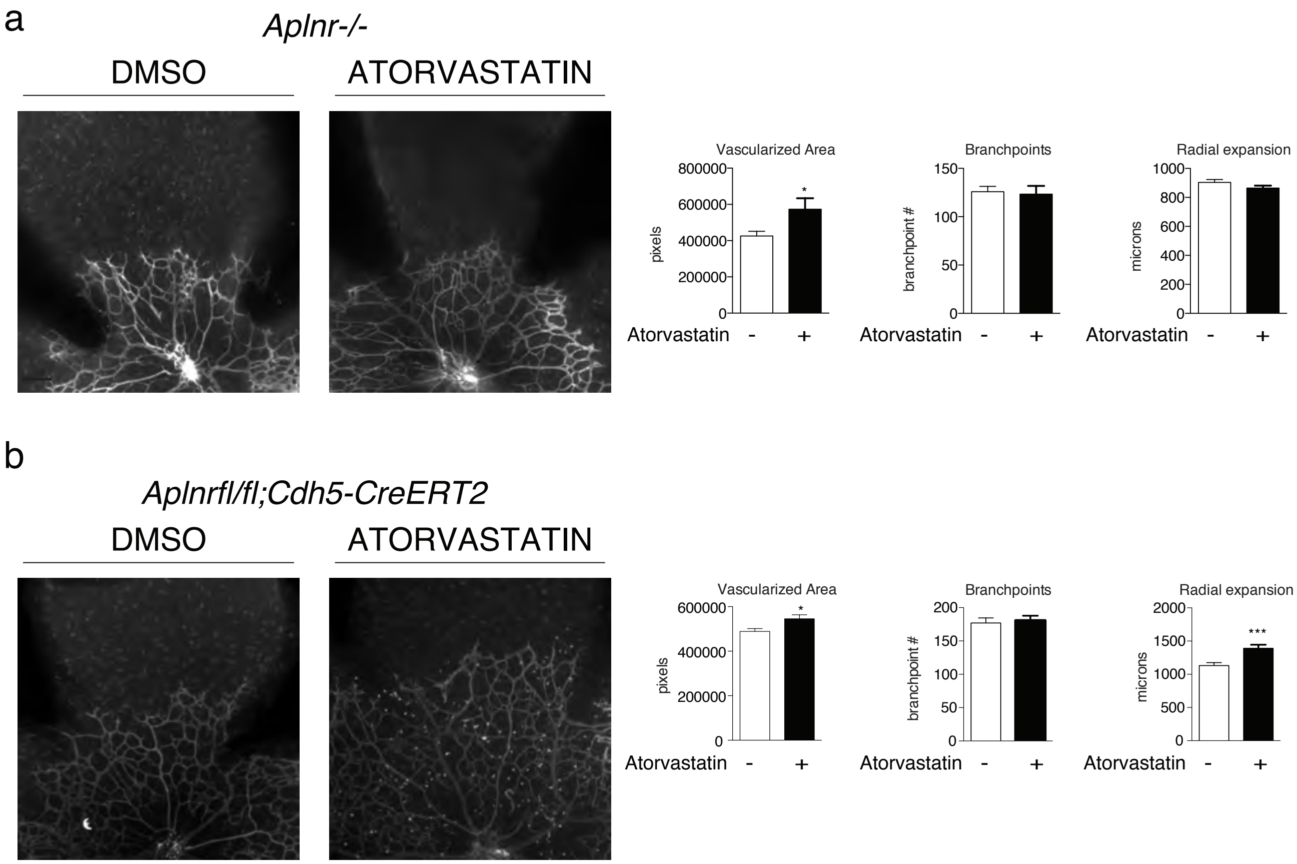

Supplementary Figure 16. Atorvastatin partially improves the *Aplnr-/-* retinal phenotypes. (a) Retinas from atorvastatin treated *Aplnr-/-* P5 pups display improved vascularized area but not number of vascular branchpoints or vascular radial growth compared to DMSO treated pups. (b) Retinas from atorvastatin treated *Aplnrfl/fl;Cdh5-CreERT2* P5 pups display improved vascularized and radial growth but not number of vascular branchpoints compared to DMSO treated pups. Scalebar, 200um. $^*P < 0.05$, $^{***}P \leq 0.001$, *t*-test. Error bars represent SEM. $n \geq 5$ retinas per genotype.

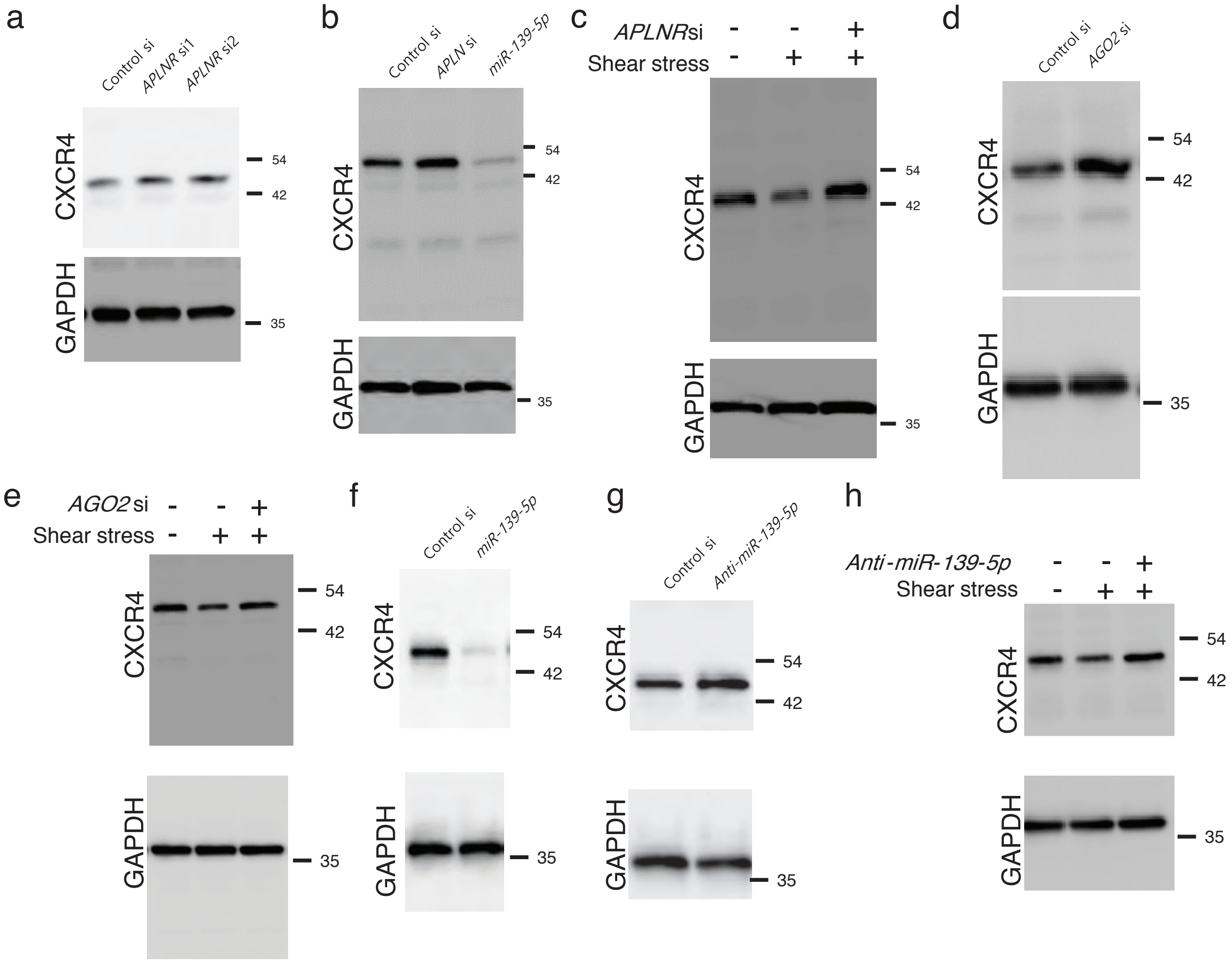

Supplementary Fig. 17. Uncroppped scans of key western blots. CXCR4 and GAPDH expression under (a) *APLNR* knockdown, (b) *APLN* knockdown, (c) *APLNR* knockdown and shear stress, (d) *AGO2* knockdown, (e) *AGO2* knockdown and shear stress, (f) miR-139-5p overexpression, (g) miR-139-5p inhibition or (h) miR-139-5p inhibition and shear stress. *n*=3 experiments per condition.
